# Predictive analysis of B-cell antigenic epitopes in phospholipase D toxins from *Loxosceles* spiders

**DOI:** 10.1016/j.toxcx.2025.100222

**Published:** 2025-03-26

**Authors:** Alejandro Catalán, Carolina García, Valentina Sambra, Nicole Cadena, José Rojas, Tomás Arán-Sekul, Juan San Francisco, Valeria Vásquez-Saez, Christian Muñoz, Abel Vásquez, Jorge E. Araya

**Affiliations:** a Laboratorio de Investigación en Parasitología Molecular, Departamento de Tecnología Médica, Facultad de Ciencias de la Salud, Universidad de Antofagasta, Antofagasta, CP 1270300, Chile; b Centro de Investigación en Inmunología y Biotecnología Biomédica de Antofagasta, Universidad de Antofagasta, Chile; c Departamento Agencia Nacional de Dispositivos Médicos, Innovación y Desarrollo, Instituto de Salud Pública de Chile, Santiago, Chile

**Keywords:** Antigenic structure, *Loxosceles* species, Epitope variation

## Abstract

The Phospholipase D (PLD) toxin family, a major component of the *Loxosceles* spider venom, is a valuable biotechnological tool for developing antivenom treatment and diagnostic assays to overcome and prevent loxoscelism. However, there is limited knowledge about the antigenic structure of the PLD family or if sequence diversity correlates with antigenic variability. This study aimed to evaluate the possible antigenic diversity of PLDs sequences among different species of spiders of the *Loxosceles* genus through a predictive analysis of potential continuous and discontinuous antigenic epitopes of two phylogenetic interspecies clusters. Thus, *L. laeta* had higher amino acid sequence variation than other species, being classified into three phylogenetic clusters at the intra-specie level. Furthermore, multiple alignments of consensus PLD sequences from each *Loxosceles* species showed two different phylogenetic clusters at interspecies level depending on the amino acid conservation. For each cluster, at least nine continuous antigenic domains were identified, and depending on the phylogenetic cluster belonging to the *Loxosceles* species, the PLD continuous and discontinuous antigenic structure varies. Also, *L. laeta* PLDs vary significantly within the *Loxosceles* species and possess their own antigenic structure compared to other species with common continuous epitopes. Finally, the catalytic loop was identified as a common discontinuous epitope in the PLDs independently of the cluster or the class it belongs to. This antigenic diversity of PLD toxins could have implications for antibody recognition and should be considered in the design strategies for the development of serum treatments and diagnostic assays to detect *Loxosceles* venom.

## Highlights

• Two interspecie phylogenetic clusters were assigned based on the conservation and phylogenetic relationship of 206 *Loxosceles* PLD sequences.
• Nine continuous antigenic epitopes were identified from the PLD toxin family of *Loxosceles* spider venom.
• PLDs from *L. laeta* showed the greatest intraspecie antigenic variability, with specific continuous antigenic epitopes.
• The active site-flanking loop of PLD showed a conserved discontinuous epitope at the tridimensional antigenic structure.

## Graphical abstract

**Figure:**
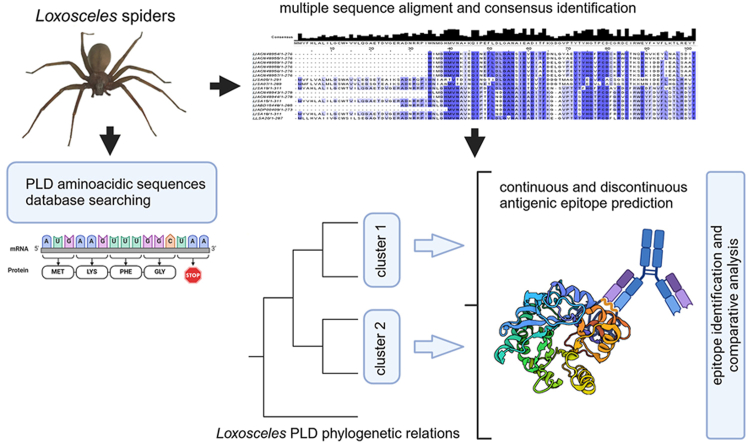

## 1 Introduction

The *Loxosceles* spider genus is composed of 147 species (World Spider Catalog, 2025). It is known to possess a highly toxic venom composed of a complex mixture of proteins and peptide toxins, including toxins expressed in large quantities, such as phospholipases-D, metalloproteases (Astacin type), and insecticidal toxins (inhibitor cysteine knot; ICK peptides), as well as toxins expressed in low quantities, such as hyaluronidases, serine proteases, serine protease inhibitors (serpins), allergenic factors, and translationally controlled tumor protein (TCTP) (Tambourgi et al., 1998; Feitosa et al., 1998; da Silveira et al., 2002; de Castro et al., 2004; Fernandes-Pedrosa Mde et al., 2008; da Silveira et al., 2007; Matsubara et al., 2013; Gremski et al., 2021a, Gremski et al., 2021b). Of all the toxic components of venom, the family of phospholipases-D (PLDs) toxins has been considered the main component of venom with deleterious effects on humans, including severe inflammatory response, edema, hemolysis, dermonecrosis, kidney, liver, and heart tissue cytotoxicity (Forrester et al., 1978; Tambourgi et al., 1998; Chaim et al., 2006, 2011; Kusma et al., 2008; Gremski et al., 2022). This kind of toxin has been expressed in 20 % up to 65.4 % of the total venom transcripts of *Loxosceles intermedia*, 71.8 % of total transcripts of *Loxosceles gaucho*, about 15 % of transcripts of *Loxosceles similis*, and between 16 % up to 69 % of the total venom transcripts of *Loxosceles laeta* (Fernandes-Pedrosa Mde et al., 2008; Gremski et al., 2010; Dantas et al., 2016; Medina-Santos et al., 2022; Theodoro et al., 2024). Studies have revealed a remarkable diversity of PLDs in *Loxosceles* spiders, regarding sequence variation and enzymatic activity, especially in species such as *L*. *intermedia* and *L*. *laeta* venoms, which contain multiple isoforms of PLDs with distinct enzymatic and biological properties (Binford et al., 2009; Gremski et al., 2020). Also, the comparative transcriptome analysis of the relevant species *L. intermedia*, *L. laeta* and *L. gaucho* corroborates the diversity intra- and inter-species of this toxin family (Theodoro et al., 2024).

Recombinant PLDs from the venom of *Loxosceles* spiders has been considered as an essential tool for biotechnological applications, with a focus on immunological responses, immunotherapy, and diagnosis of loxoscelism, since the recombinant PLD proteins can be used as an antigen to generate monoclonal antibodies or even monospecific polyclonal antibodies, which, in the diagnosis of *Loxoscelism*, would be useful for ELISA assays and lateral flow immunochromatography assays (LFIA), and in immunotherapy would be beneficial to overcome the low availability of venom antigens for antivenom production, since recombinant toxins can supply sufficient material for the antigen, and the antibodies obtained against them, can originate molecules with high affinity, avidity and greater interaction strength of interaction with the antigens, and neutralization capacity, which results in valuable tools for diagnosis and treatment (Fingermann et al., 2020; Senff-Ribeiro et al., 2008). Despite the recognized and remarkable intra- and inter-species diversity about sequence variation and enzymatic activity in the PLD toxins family from *Loxosceles* spiders, the venoms have shown a significant cross-reactivity, especially at sera produced against geographically related *Loxosceles* species. As have been showed in venoms of *Loxosceles* spiders present in North America, such as *L. reclusa* and *L. deserta,* which possess venoms with a similar venom banding pattern and a marked cross-detection with polyclonal sera produced against each venom (Gomez et al., 2001). This cross-reactivity between polyclonal sera was also reported between the South American species *L.* *gaucho*, *L*. *intermedia*, and *L*. *laeta*; however, from the three species, the lower titers were shown with serum produced against *L. laeta* venom (Barbaro et al., 1994). This could explain some antigenic variations that could be seen when different *Loxosceles* species have been exposed to commercial antisera produced against *Loxosceles* venoms, such as the anti-arachnid serum (SAA) produced against *L. gaucho* and other spider venoms, or the poly-specific anti-*Loxosceles* serum (PSLAS) produced against equal parts of the venoms of *L. gaucho*, *L. laeta*, and *L. intermedia*, where the SAA was able to cross-detect the venoms of *Loxosceles* species *L*. *deserta*, *L. gaucho*, *L. intermedia*, and *L. reclusa*; however, again the detection titer of the *L. laeta* venom was significantly lower in relation to the other venoms. Meanwhile, the PSLAS serum showed equal cross-detection capacity for all the venoms tested (Barbaro et al., 2005). The latter points toward possible antigenic differences of the *L. laeta* venom in relation to the rest of the *Loxosceles* genus. In this sense, it has been reported that monoclonal antibodies produced against the 35 kDa dermonecrotic component of *L. gaucho* venom have a low or almost no detection capacity against *L. laeta* venom and the 32 kDa protein component (phospholipases-D) (Guilherme et al., 2001). In addition, cross-neutralization is not always observed among antibodies produced against PLDs from all *Loxosceles* species (Gremski et al., 2021a, Gremski et al., 2021b), as, for example, the anti-serum against PLD from *L. intermedia* produces only a partial protection against animals exposed to the Peruvian *L*. *laeta* venom (Duarte et al., 2015). Also, comparative detection of polyclonal serum produced against recombinant PLD from *L. laeta*, *L. intermedia*, and *L. gaucho* showed cross-reactivity, but its detection was lower when *L. laeta* anti-PLD serum was incubated with *L. intermedia* PLD antigen and vice versa when *L. intermedia* anti-PLD serum was put in contact with *L. laeta* PLD antigen (da Justa et al., 2023). The above led us to consider the presence of relevant inter-species antigenic variations between the venoms of *Loxosceles* species and those associated specifically with the PLD family toxins. These antigenic differences of the PLD toxins have already been reported at the intra-species level, where the cross-reactivity of an anti-venom serum produced against three different recombinant PLD toxins from *L. intermedia* showed differential capacity to detect other isoforms, this detection being greater against the recombinant antigen LiRecDT1 but significantly lower against the antigens LiRecDT2 and LiRecDT3 (Ribeiro et al., 2007).

The antigenic structure of phospholipase-D from *Loxosceles* spiders is, however, poorly understood and characterized, since the most studies performed to identify possible antigenic epitopes have only focused on single recombinant isoforms of PLD, such as recLiD1 of *L. intermedia* (Kalapothakis et al., 2002; Felicori et al., 2006) and SMase I of *L. laeta* (Fernandes et al., 2002), and have not considered the possibility of significant amino acid sequence variation amongst *Loxosceles* species. Thus, the lineal epitope NLGANSIETDVSFDDNANPEYTYHGIP (residues 25–51) of the N-terminal region of the recombinant protein recLiD1 of *L. intermedia* was reported to be detected by anti-recLiD1 serum and was also capable of generating protective antibodies against lethal and dermonecrotic effects in mice treated with *L. intermedia* venom (Felicori et al., 2006); in addition, the sera produced against this peptide showed cross-detection against the venoms of *L. gaucho*, *L. laeta* (Brazil), and *L. laeta* (Peru) (Dias-Lopes et al., 2010). Subsequently, using the same LiD1 protein, the peptides MVNAIGQIDEFVNLG, IETDVSFDDNANPEY, SKKYENFNDFLKGLR, DNQANDAGKKLAKNL, DKVGHDFSGNDDISD, and NYPDVITDVLNEAAY were identified and detected by the polyclonal anti-recLiD1 serum. However, only the epitope SKKYENFNDFLKGLR showed a strong immune response, while the others were not immunogenic (Felicori et al., 2009). Also, the peptides DNRRPIWNLAHMVNA (residues 2–16) and DFSGPYLPSLPTLDA (residues 164–178) of SMase I from *L. laeta*, and the peptide EFVNLGANSIETDVS (residues 22–36) at A1H-LoxGa from *L. gaucho* and LiD1 from *L. intermedia* were identified and showed several differences, since the peptides from *L. laeta* possess 73 % and 27 % identity with their respective sequences in the species *L. gaucho* and *L. intermedia,* while the epitope from these species showed only 53 % identity with the *L. laeta* PLD antigen (Ramada et al., 2013). In this way, it is possible to hypothesize a significant antigenic variation between PLD isoforms depending on the *Loxosceles* species. Hence, a comparative characterization of these antigenic epitopes by species seems to be necessary. However, a detailed analysis of the presence of B-cell antigenic epitopes in the family of phospholipase D toxins for each species of *Loxosceles* spiders has not been performed. The goal of this study was to perform a predictive analysis of potential continuous and discontinuous antigenic epitopes for the family of phospholipase D toxins from *Loxosceles* spiders, and the importance of its intra- and inter-species variations on the production of monoclonal antibodies with potential application in diagnostic assays and the preparation of specific antisera for treatment are discussed.

## 2 Materials and methods

### 2.1 Phospholipase D sequences

A search for nucleotide sequences and the corresponding protein sequences of phospholipases D from spiders of the *Loxosceles* genus was performed using the National Center for Biotechnology Information (NCBI) website, and four different keywords were used according to the nomenclature used for these toxins in the literature. Thus, the search 1, includes the terms “Sphingomyelinase D″ AND “*Loxosceles”* [orgn]; the search 2: “Phospholipase D” AND “*Loxosceles”* [orgn]; the search 3: “Dermonecrotic Toxin” AND “Loxosceles” [orgn]; and the search 4: “LoxTox Protein” AND “*Loxosceles”* [orgn]. If available, the complete nucleotide sequences (between 700 and 4000 bp) and protein sequences of phospholipases D in FASTA format for each *Loxosceles* species were copied to a file. The selection of nucleotide sequences with a length larger than 700 bp was considered based on the previously reported average length of the *L. intermedia* transcriptome cDNAs (Gremski et al., 2010) and the complete length of the *L. laeta* PLD cDNA (Catalan et al., 2011). When the protein sequence access number was not available for its corresponding nucleotide sequences, then a search of the open reading frame (ORF) sequence using the tool ORFinder (https://www.ncbi.nlm.nih.gov/orffinder/) was carried out. Thus, the amino acid sequences were selected considering the different ORFs according to the sequence with the largest length of base pairs and of the positive sense strand, and then a protein identification number was assigned, consisting of the first letters of the genus and species (e.g., *Loxosceles laeta*; Ll), followed by the letters AAS (Amino Acid Sequence) and the correlative number according to species (001, 002, …n).

### 2.2 Multiple sequence alignment for phospholipase D sequences

The amino acid sequences for phospholipases D from each *Loxosceles* species were aligned using the software Clustal Omega multiple sequence alignment (https://www.ebi.ac.uk/Tools/msa/clustalo/) (Sievers et al., 2011). Thus, each PLD amino acid sequence in FASTA format was submitted using the default parameters, and the alignment results were visualized and edited to identify conserved amino acids (100 % identity) using the software Jalview (https://www.jalview.org/) (Waterhouse et al., 2009). Also, the percentage identity matrix data for each *Loxosceles* species was analyzed and compared. Following this, the obtained amino acid consensus sequence for each *Loxosceles* species was identified. Depending on the *Loxosceles* species analyzed, when the identity percent showed a highly conserved sequence (higher than 60 up to 80 % identity percent), only a consensus sequence was considered, but when the identity percent showed a lower sequence conservation (lower than 50 % identity percent), two or more consensus sequences were assigned by the *Loxosceles* species and identified as “consensus 1, 2, …n.”. Additionally, the identified amino acid consensus sequences by *Loxosceles* species were used for multiple sequence alignment at the genus level using Clustal Omega software and visualized in Jalview.

### 2.3 Phylogenetic analysis for PLD aminoacidic sequences

The evolutionary analysis for PLD amino acid sequences from each *Loxosceles* species and for PLD consensus sequences at the genus level was inferred by using the Maximum Likelihood method and the Whelan and Goldman model (Whelan and Goldman, 2001) in the MEGA X software (Kumar et al., 2018). Thus, each phylogenetic tree was constructed using the highest log likelihood data, using the Neighbor-Joining method, with a consensus tree inferred from 1000 Bootstrap replicates and evolutionary distances were computed using the Maximum Composite Likelihood method. A discrete Gamma distribution was used to model evolutionary rate differences among sites for each phylogenetic tree constructed. Clustered sequences were shown in brackets. The percentage of trees in which the associated taxa clustered together was displayed next to the tree branches. Sequences were clustered considering the identity percentage over 80 %, 60 %, and 50 %. Also, the software MMSeqs2 was used to corroborate the clustering of sequences in the phylogenetic tree (Steinegger and Soding, 2017; Zimmermann et al., 2018).

### 2.4 Continuous and discontinuous antigenic epitope analysis

Continuous and discontinuous antigenic sequences were inferred for each *Loxosceles* species using the PLD consensus amino acid sequences identified without including the signal peptide sequence at the N-terminal. Thus, three online available continuous antigenic epitope predictors were used to identify antigenic domains. The predictors used were Bepipred linear epitope prediction 2.0 (http://tools.iedb.org/bcell/) (Jespersen et al., 2017), using default parameters and a threshold assigned at 0.5; BEPITOPE (bepitope.ibs.fr) (Odorico and Pellequer, 2003), using default parameters and a threshold assigned at 0.5; and ABCpred epitope prediction server (https://webs.iiitd.edu.in/raghava/abcpred/) (Saha and Raghava, 2006), using a threshold score for linear epitopes assigned at 0.7. The assignation of a peptide region as a possible antigenic epitope was assigned considering the overlapping position of findings from the three separate B-cell epitope prediction softwares (Bepipred 2.0, BEPITOPE, and ABCpred). Thereby, a PLD amino acid sequence was considered to have a potential antigenic domain when it was recognized by all three predictors and found to be present in the majority of the aligned PLD consensus sequences. Additionally, the consensus amino acid residues obtained from the multiple alignments of PLDs consensus sequence from each *Loxosceles* species were used to determine the amino acid sequence of each linear antigenic epitope. When a majority could not be achieved for a particular amino acid residue, the given amino acid residue was chosen over other possible amino acids based on the one that has the highest antigenic score over the threshold when analyzed by the predictor Bepipred 2.0.

Additionally, the presence of conformational antigenic epitopes in PLD consensus sequences was analyzed using the server ElliPro: antibody epitope prediction (http://tools.iedb.org/ellipro/) (Ponomarenko et al., 2008) using a threshold score of 0.6. To perform the prediction, the tridimensional PLD structure was necessary to perform the prediction since the ElliPro epitope predictor uses a PDB file to predict the conformational epitope, so the needed PLD tridimensional structure was modeled. Thus, only the PLD amino acid consensus sequences for the *Loxosceles* species *L*. *laeta* and *L. intermedia* were tridimensionally modeled, since the only available X-ray obtained PDB templates for the homology modeling were the ones corresponding to the species *L. laeta* and *L. intermedia*. Thus, for *L. laeta*, the template used was the Sphingomyelinase D1 from *L. laeta* (PDB: 2F9R) (Murakami et al., 2005). For *L*. *intermedia*, the template used was the Phospholipase D class II from *L. intermedia* (PDB: 3RLH) (de Giuseppe et al., 2011). The PLD 3D structure was inferred using the SWISS-MODEL Workspace server available at https://www.expasy.org/resources/swiss-model-workspace (Guex and Peitsch, 1997). Also, the predictor CBTOPE: prediction of conformational B-cell epitopes (http://crdd.osdd.net/raghava/cbtope/submit.php) (Ansari and Raghava, 2010) was used to identify amino acid residues as possible conformational antigenic epitopes since the program use the linear amino acid sequence in FASTA format for the analysis. Finally, the modeled 3D structures of PLD were visualized and edited using the DeepView/Swiss-PdbViewer program v4.1 (http://www.expasy.org/spdbv/).

## 3 Results

### 3.1 Amino acid sequences and phylogenetic analysis of phospholipases D from *Loxosceles* spiders

Based on the established nomenclature for the phospholipases-D (PLD) toxins, we considered four keywords for searching for PLD from the venom of *Loxosceles* spiders in the National Center for Biotechnology Information (NCBI) database. The keywords used were “Sphingomyelinase D″, “Phospholipase D″, “Dermonecrotic Toxin”, and “LoxTox Protein”. The initial search, using the term “Sphingomyelinase D″, showed 225 nucleotide sequences. However, many of the sequences displayed were partial sequences. Therefore, only complete sequences and those whose base pair lengths varied from 700 to 4000 bp were included. Thus, there were 178 sequences selected; these sequences corresponded to nucleotide sequences of the following *Loxosceles* species: *L. adelaida*, *L. amazonica*, *L. apachea*, *L. arizonica*, *L. boneti*, *L. deserta*, *L. gaucho*, *L. hirsuta*, *L. laeta*, *L. reclusa*, *L. rufescens*, *L. sabina*, *L. spadicea*, *L. spinulosa*, *L. variegata*, and *L.* spp. Among these, the species *L. laeta*, *L. hirsuta*, and *L. deserta* had the largest number of sequences. During this search, no *L. intermedia* sequences were found, and the two sequences for unknown species were excluded. Followed, using the term “phospholipase D″ for the second search, eight sequences corresponding to the nucleotide sequences of the *Loxosceles* species: *L. laeta*, *L. gaucho*, *L. intermedia*, *L. reclusa*, and *L. arizonica*, were found. From them, only one of the sequences from *L. gaucho* was taken into consideration after excluding the repeated sequences found in the initial search. Also, nine sequences were found in the third search using the keyword “Dermonecrotic Toxin”. These sequences were the nucleotide sequences of *L. intermedia* (7 seq), *L. laeta* (1 seq), and *L. similis* (1 seq). From these, the *L. laeta* sequence was excluded due to its sequence length being shorter than 700 bp, and two of the seven *L. intermedia* sequences were duplicates found in the second search. Using the keyword “LoxTox Protein” in the fourth search, we found 48 sequences that matched the nucleotide sequences of two *Loxosceles* species: *L. intermedia* (14 seq) and *L. similis* (34 seq). Out of these, only 25 out of the 34 *L. similis* sequences were included based on the length exclusion criterion, and seven *L. intermedia* sequences were considered since the remaining seven sequences had already been included in the second and third searches. Additionally, the sequences of *L. laeta*′s SMase I (Protein Access N°: AAM21154.1) and *L. intermedia′s* LiD1 (Protein Access N°: P0CE81) were also included, since these sequences were not found in any of the prior four searches. Ultimately, 206 amino acid sequences of PLD from different *Loxosceles* species were considered. Despite the most known *Loxosceles* species were included in the study, this was only 12 % of species with amino acid sequences for PLD that are available in the NCBI database from the 147 *Loxosceles* species cataloged in the World Spider Catalog for the genus *Loxosceles* (*Sicariidae*).

**Fig. 1:**
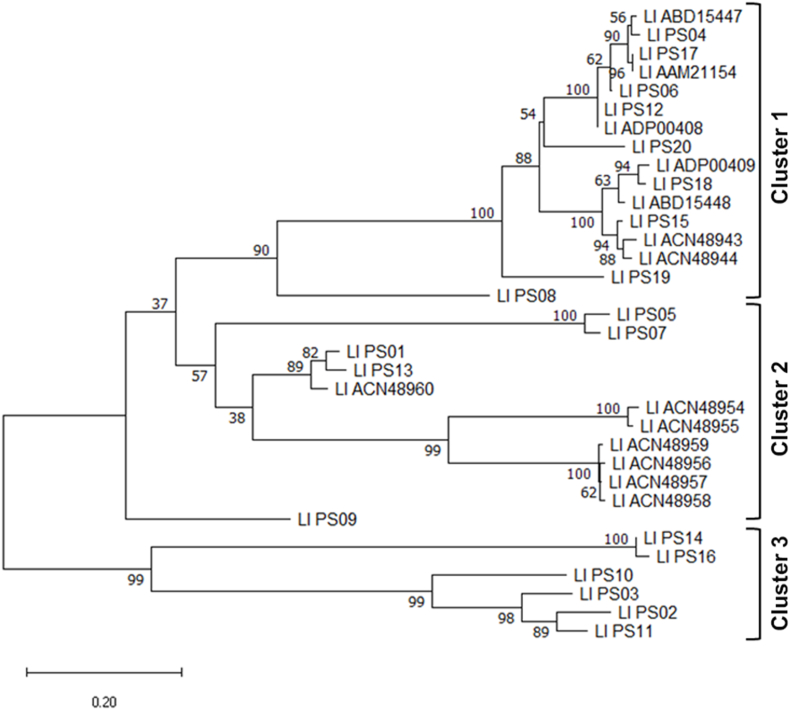
Intra-specie phylogenetic tree of *Loxosceles laeta* phospholipases D. Maximum likelihood phylogenetic tree of amino acid sequences of phospholipases D from *L. laeta*. The tree was inferred by using the Maximum Likelihood method and Whelan and Goldman model, and the tree with the highest log likelihood (−6478.97) is shown. The bootstrap method was used as a phylogeny test for 1000 bootstrap replicates. The percentage of trees in which the associated taxa are clustered together is displayed next to the branches. A discrete Gamma distribution was used to model evolutionary rate differences among sites (5 categories (+G, parameter = 1.6811)). This analysis involved 34 amino acid sequences. There were a total of 334 positions in the final dataset. Evolutionary analyses were conducted in MEGA X.

Following, the amino acid sequences of PLDs from each species of *Loxosceles* were multiple aligned to identify a specie-representative consensus amino acid sequence of PLD, which would be further analyzed to determine the presence of antigenic epitopes. Because of the high heterogeneity found between the amino acid sequences of the available PLD isoforms, it was necessary to consider more than one consensus sequence in some *Loxosceles* species. Thus, the alignment and followed phylogenetic analysis of the 34 sequences of *L. laeta* (percent identity ranging from 42.38 to 100 %) showed three phylogenetic intra-specie clusters of sequences (Fig. 1), and a total of five possible consensus sequences were identified (Supplementary Fig. 1). The same was observed with the PLD sequences of *L. intermedia,* where from the 15 sequences considered (percent identity ranged from 43.56 to 99.67 %) two phylogenetic intra-specie clusters and a total of two consensus sequences were identified (Supplementary Fig. 2). Also, the alignment and followed phylogenetic analysis of the 25 sequences of *L. similis* (identity percentage ranging from 32.31 to 99.68 %) showed two phylogenetic intra-specie clusters, and a total of three consensus sequences were identified (Supplementary Fig. 3). Additionally, it was possible identify more than one PLD consensus sequence in other *Loxosceles* species, as *L. arizonica* [15 sequences; identity percentage ranged from 43.01 to 99.27 %; two phylogenetic intra-specie clusters; two consensus sequences identified] and *L. spinulosa* [15 sequences; identity percentage ranged from 38.48 to 99.64 %, three intra-specie phylogenetic clusters; two consensus sequences identified]. Meanwhile, other groups of *Loxosceles* species, formed by species *L. amazonica*, *L. apachea*, *L. hirsuta*, *L. reclusa*, *L. sabina*, *L. spadicea*, *L. variegata*, *L. deserta*, and *L. rufescens*, displayed a significant grade of similarity in their PLD amino acid sequences, so it was possible to identify only one PLD consensus sequence, except for the species *L. deserta* and *L. rufescens*, where two consensus sequences were considered for each. Also, since *L. boneti* and *L. gaucho* have only two sequences available for each, and considering that the identity percentage between the two sequences was between 40 and 50 % in both species, no consensus could be found in each case. Consequently, both sequences of each species were used for epitope analysis. Finally, since *L. adelaida* has only one PLD sequence available, no consensus was considered.

**Fig. 2:**
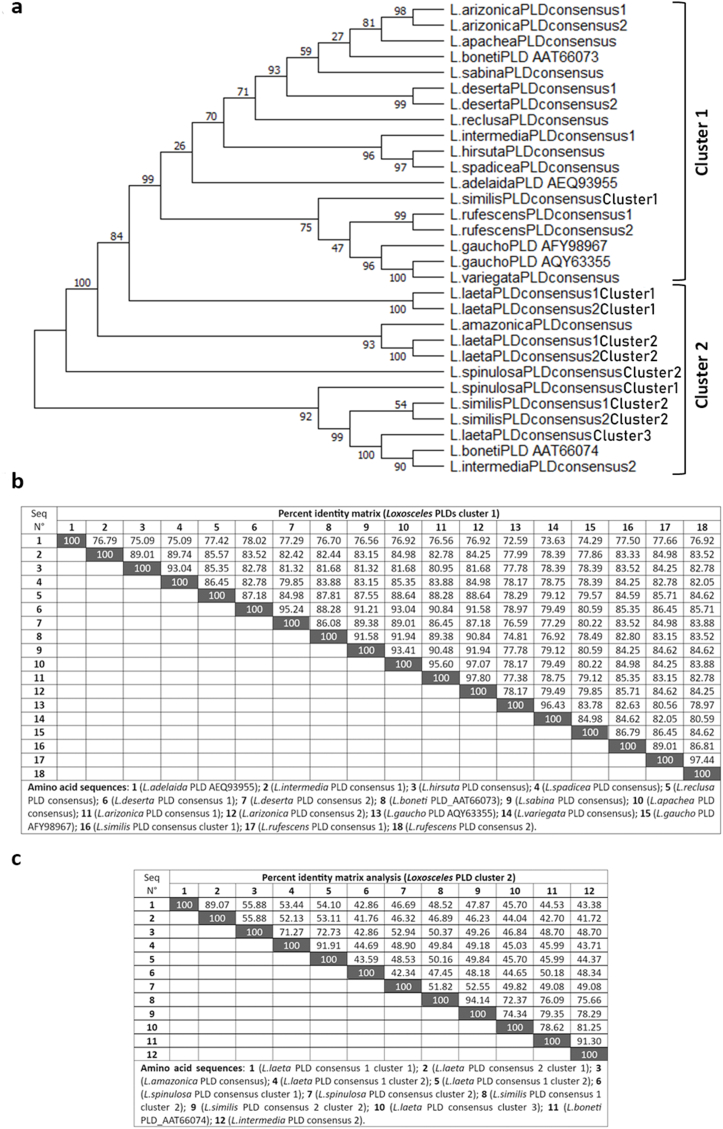
Phylogenetic analysis of consensus phospholipases D sequences from spider of *Loxosceles* genus. **a**). Maximum likelihood phylogenetic tree of aminoacidic consensus sequences of phospholipases D from *Loxosceles* spiders. The tree was inferred by using the Maximum Likelihood method and the Whelan and Goldman model, and the tree with the highest log likelihood (−6809.57) is shown. The percentage of trees in which the associated taxa are clustered together is displayed next to the branches. Initial tree(s) for the heuristic search were obtained automatically by applying Neighbor-Join and BioNJ algorithms to a matrix of pairwise distances estimated using the JTT model. Then selecting the topology with superior log likelihood value was selected. A discrete Gamma distribution was used to model evolutionary rate differences among sites (5 categories (+G, parameter = 1.3139)). This analysis involved 30 amino acid sequences. There was a total of 350 positions in the final dataset. Evolutionary analyses were conducted in MEGA X. The tree is shown only as a topology (without scale bar) to facilitate the visualization of the clusters. **b**) Percent identity matrix of aminoacidic consensus sequences of phospholipases D from *Loxosceles* phylogenetic inter-specie cluster 1. Percent identities were obtained for 18 consensus sequences using the Clustal Omega multiple sequence alignment software v.1.2.4. **c)** Percent identity matrix of aminoacidic consensus sequences of phospholipases D from *Loxosceles* phylogenetic inter-specie cluster 2. Percent identities were obtained for 12 consensus sequences using the Clustal Omega multiple sequence alignment software v.1.2.4. Consensus sequences are listed as table foot.

When the consensus sequences of PLDs from *Loxosceles* species were analyzed and a phylogenetic tree was constructed, the inter-species variability of PLD isoforms was further demonstrated. As shown in Fig. 2, the *Loxosceles* genus was found to have two inter-species phylogenetic clusters. One of these clusters included the following species: *L. arizonica*, *L. apachea*, *L. boneti* (AAT66073), *L. sabina*, *L. deserta*, *L. reclusa*, *L. hirsuta*, *L. spadicea*, *L. rufescens*, *L. adelaida*, *L. gaucho*, *L. variegata*, *L. similis* consensus cluster 1, and *L. intermedia* consensus 1. While the *Loxosceles* species: *L. amazonica*, *L. spinulosa*, *L. boneti* (AAT66074), *L. similis* (consensus 1 and 2 of intra-specie cluster 2), *L. intermedia* consensus 2, and *L. laeta* (consensus from intra-specie clusters 1, 2, and 3) constitute the second *Loxosceles* inter-specie phylogenetic cluster. The species of *Loxosceles* belonging to the latter cluster have consensus sequences of PLD that are less conserved than those of the other species in *Loxosceles* inter-specie phylogenetic cluster 1.

### 3.2 Antigenic epitope analysis for the PLD of *Loxosceles*

**Fig. 3:**
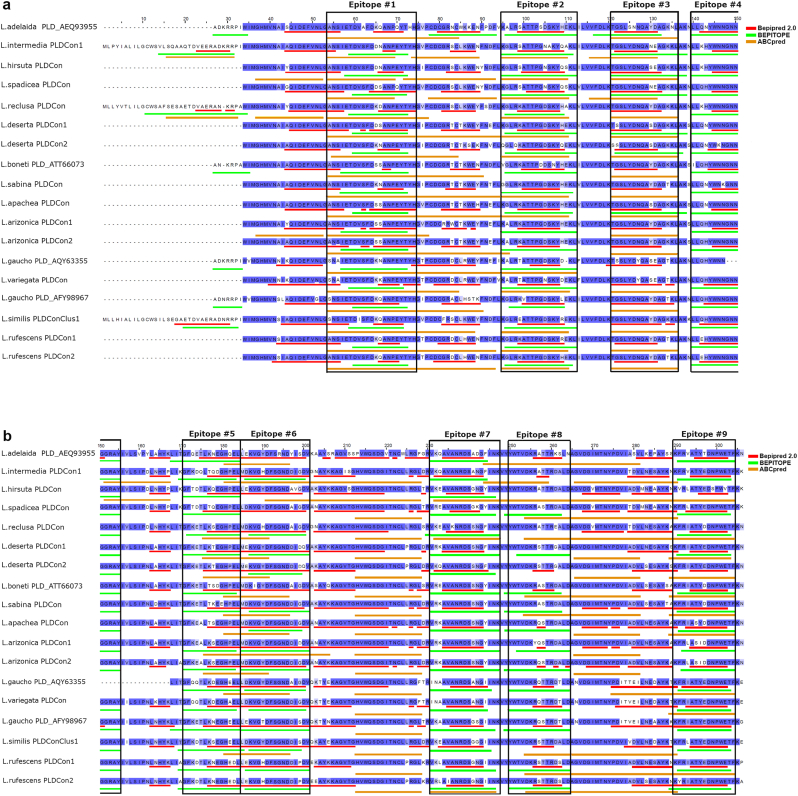
Antigenic domains for continuous epitopes of phospholipases D from inter-specie phylogenetic cluster 1 of *Loxosceles* genus. PLD consensus sequences were multiple aligned using the Clustal Omega multiple sequence alignment software v.1.2.4., and the image was edited using Jalview software. Linear epitope predictors used are indicated in order as color lines below the sequences. Red: Bepipred 2.0, green: BEPITOPE, orange: ABCpred. Antigenic domains are shown in black boxes. PLD identity percentages of amino acid residues are shown with colors according to their grade of identity. blue: >80 % identity; light blue: 80-60 % identity; sky-blue: <50 % identity. **a)** N-terminal analysis. **b)** C-terminal analysis. (For interpretation of the references to color in this figure legend, the reader is referred to the Web version of this article.)

**Fig. 4:**
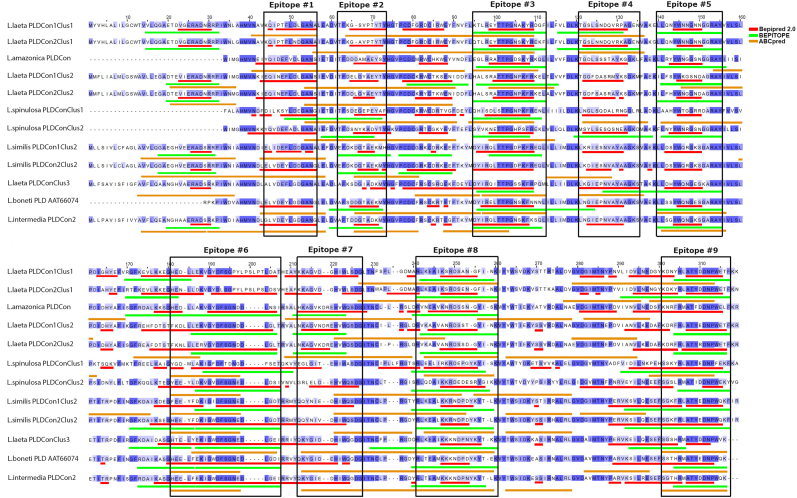
Antigenic domains for continuous epitopes of phospholipases D from inter-specie phylogenetic cluster 2 of *Loxosceles* genus. PLD consensus sequences were multiple aligned using the Clustal Omega multiple sequence alignment software v.1.2.4., and image was edited using Jalview software. Linear epitope predictors used are indicated in order as color lines below the sequences. Red: Bepipred 2.0, green: BEPITOPE, orange: ABCpred. Antigenic domains are shown in black boxes. PLD identity percentages of amino acid residues are shown with colors according to their grade of identity. blue: >80 % identity; light blue: 80-60 % identity; sky-blue: <50 % identity. (For interpretation of the references to color in this figure legend, the reader is referred to the Web version of this article.)

**Fig. 5:**
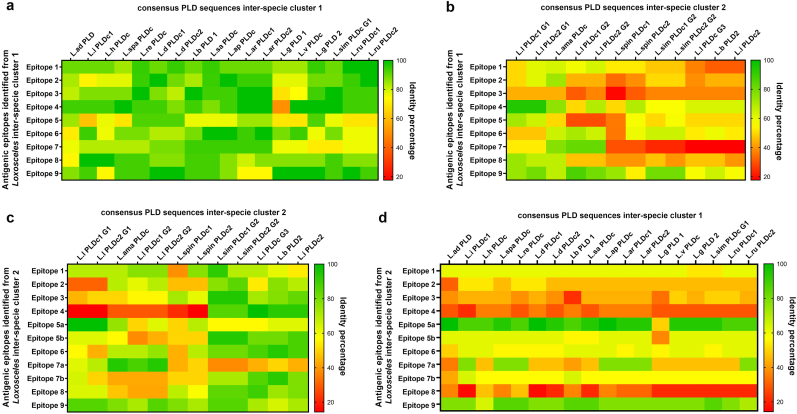
*Loxosceles* species continuous antigenic epitope's identity percentage heat map. **A, b).** The nine identified epitope sequences from species of *Loxosceles* inter-specie phylogenetic cluster 1 were compared at the identity percentage with each PLD consensus sequence of *Loxosceles* from inter-specie phylogenetic cluster 1 (**a**) or PLD consensus sequence of *Loxosceles* from inter-specie phylogenetic cluster 2 (**b**). Heat map color was assigned as the higher percentage of identity (green) or the lower percentage of identity (red). **C, d**). The nine identified continuous antigenic epitopes from species of *Loxosceles* inter-specie phylogenetic cluster 2 were also compared at the identity percentages with each PLD consensus sequence of *Loxosceles* from inter-specie phylogenetic cluster 1 (**c**) or PLD consensus sequence of *Loxosceles* from inter-specie phylogenetic cluster 2 (**d**). (For interpretation of the references to color in this figure legend, the reader is referred to the Web version of this article.)

The amino acid consensus sequences of PLD from the *Loxosceles* species were analyzed to predict the presence of continuous (linear) antigenic epitopes. Thus, at least nine antigenic domains could be found for the PLD sequences from the *Loxosceles* genus belonging to the inter-specie phylogenetic cluster 1 (Fig. 3), whose amino acid sequences are listed in Table 1. Also, PLD consensus sequences from *Loxosceles* species belonging to the inter-specie phylogenetic cluster 2 displayed the nine antigenic domains, similar to sequences from cluster 1, but with reduced conservation of amino acid residues between them (Fig. 4). Table 2 lists the antigenic epitopes found for this cluster. Most of the predicted continuous antigenic epitopes were cluster-specific, except for the epitopes LLQNYWNNGNNGGRAY and FRLATYDDNPWEKF, which were found in consensus sequences of PLD from the two phylogenetic clusters of *Loxosceles* and therefore were considered as common epitopes. The identity percentage of the amino acid sequences of the nine continuous antigenic epitopes identified for each *Loxosceles* inter-specie phylogenetic cluster was then compared by *Loxosceles* species to determine its conservation. Thus, most of the predicted epitopes identified from the PLD consensus sequences belonging to the inter-specie phylogenetic cluster 1, showed a high grade of conservation between the *Loxosceles* species of the same cluster (Fig. 5a), while the percentage of identity decay significantly when compared with sequences belonging to the inter-specie phylogenetic cluster 2 (Fig. 5b), except for the epitopes #4 (LLQNYWNNGNNGGRAY) and #9 (FRLATYDDNPWEKF), which were more conserved and could be considered as common epitopes, in contrast of the epitopes #3 (TGSLYDNQAYDAGKKL) and #7 (VRQAVANRDSSNGYINK) which were completely absent. About the predicted epitopes identified from the PLD consensus sequences belonging to the inter-specie phylogenetic cluster 2, the identity percentage showed a lower conservation of epitopes sequences when compared with the consensus PLD sequences of the same cluster (Fig. 5c), where the epitope #4 (LKGIEPNVAYAGKS) was completely absent in sequences from *L. laeta*, *L. spinulosa*, and *L. amazonica*. Meanwhile, when the epitopes were compared at the identity percentage with consensus sequences belonging to the inter-specie phylogenetic cluster 1, it was possible to find a more significant variation of epitopes, where the epitopes #2 (EFDDDGTAEYMYH), #3 (DYIRQLTTPGNPKFREQL), #4 (LKGIEPNVAYAGKS), and #8 (RLKEAIKKRDDPNYKYTLK) were entirely absent (Fig. 5d). However, the epitopes LLDSYWQNGKSGARAY and FRLATYDDNPWEKF are conserved between sequences belonging to cluster 1 and 2, and could be considered as common epitopes. Because of this unique epitope variation from species of the inter-specie phylogenetic cluster 2, next we thoroughly examine the existence of unique antigenic epitopes in *L. laeta*, which was the species exhibiting the highest intraspecies variation. Thus, it was possible to identify at least six unique continuous antigenic epitopes for *L. laeta* that were absent from PLD consensus sequences from other *Loxosceles* species (Table 3). Some of these epitopes were intra-specie cluster-specific within the *L. laeta* PLD sequences. Additionally, the PLD consensus sequences of *L. laeta* revealed the absence of linear antigenic epitopes previously identified from other species of *Loxosceles* from inter-specie clusters 1 and 2, and were considered as specific for PLDs from *L. laeta*, such as the epitope LKGIEPNVAYAGKS, and VRQAVANRDSSNGYINK. Thus, in contrast to PLDs from the other species in the *Loxosceles* genus, this last finding suggests a distinctive antigenic structure for PLDs from *L. laeta*.

**Table 1:** Summary of linear antigenic epitopes identified in amino acid sequences of phospholipases D from inter-specie phylogenetic cluster 1 of *Loxosceles* spiders.

N°	Amino acid sequence^a^	Length^b^	Common epitope/cluster specific epitope
**1**	ANSIETDVSFDKQANPEYT	19	Specific for cluster 1
**2**	KGLRKATTPGDSKYHEKL	18	Specific for cluster 1
**3**	TGSLYDNQAYDAGKKL	16	Specific for cluster 1
**4**	LLQHYWNNGNNGGRAY	16	Common for cluster 1 and 2
**5**	GFKETLKNEGHPEL	14	Specific for cluster 1
**6**	MDKVGYDFSGNDDIGDV	17	Specific for cluster 1
**7**	VRQAVANRDSSNGYINK	17	Specific for cluster 1
**8**	YWTVDKRATTRDALD	15	Specific for cluster 1
**9**	KFRIATYEDNPWET	15	Common for cluster 1 and 2

a Epitope sequence identified following using the antigenic epitope predictors Bepipred 2.0, BEPITOPE, and ABCpred.

b Length of the antigenic epitope according to the number of amino acid residues that make it up.

**Table 2:** Summary of linear antigenic epitopes identified in amino acid sequences of phospholipases D from inter-specie phylogenetic cluster 2 of *Loxosceles* spiders.

N°	Amino acid sequence^a^	Length^b^	Common epitope/cluster specific epitope
**1**	EQIDEFLDDGANA	13	Specific for cluster 2
**2**	EFDDDGTAEYMYH	13	Specific for cluster 2
**3**	DYIRQLTTPGNPKFREQL	18	Specific for cluster 2
**4**	LKGIEPNVAYAGKS	14	Specific for cluster 2
**5A**	LLQNYWNNGNNGGRAY	16	Common for cluster 1 and 2
**5B**	LLDSYWQNGKSGARAY	16	Common for cluster 1 and 2
**6**	EKIGWDFSGNEDLGDI	16	Specific for cluster 2
**7A**	KKAGVKDDREHVWQSDG	16	Specific for cluster 2
**7B**	YQKYGIDEHIWQSD	14	Specific for cluster 2
**8**	RLKEAIKKRDDPNYKYTLK	19	Specific for cluster 2
**9**	FRLATYDDNPWEKF	14	Common for cluster 1 and 2

a Epitope sequence identified following using the antigenic epitope predictors Bepipred 2.0, BEPITOPE, and ABCpred.

b Length of the antigenic epitope according to the number of amino acid residues that make it up.

**Table 3:** Specific linear antigenic epitopes identified in amino acid sequences of phospholipases D from *Loxosceles laeta.*

N°	Amino acid sequence^a^	Length^b^	*L. laeta* cluster specific epitope
**1**	TGALSNDQVRPAGENV	16	*L. laeta* cluster 1
**2**	GFKEVLKKEGHEDLLEK	17	*L. laeta* cluster 1
**3**	FREAFDTSTKKDLL	14	*L. laeta* cluster 2
**4**	CDTGRDCIRWEYF	13	*L. laeta* cluster 1
**5**	CDCKRWCTKSENIDDF	16	*L. laeta* cluster 2
**6**	IKKKNDPNYKYTKK	14	*L. laeta* cluster 3

^a^Epitope sequence identified following using the antigenic epitope predictors Bepipred 2.0, BEPITOPE, and ABCpred.^b^ Length of the antigenic epitope according to the number of amino acid residues that make it up.

**Fig. 6:**
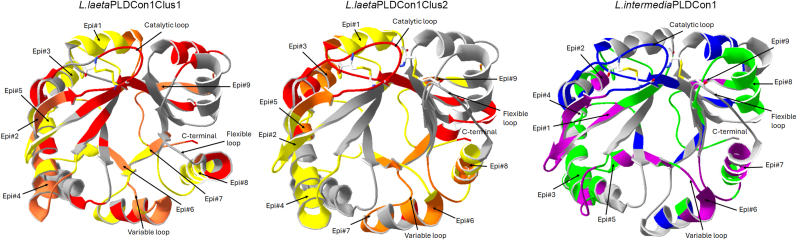
Comparison of continuous and discontinuous antigenic structure between *L. laeta* and *L. intermedia* PLDs. The modeled tertiary structure for the consensus PLD sequences of *L. laeta* (L.laetaPLDcon1clus1 and L.laetaPLDcon1clus2) and *L. intermedia* (L.intermediaPLDcon1) was visualized using Swiss-Pdbviewer software. For L. *laeta* PLDs, amino acid residues that are part of continuous epitopes are shown in yellow, while amino acid residues that are part of discontinuous epitopes are shown in red. Amino acid residues that are part of both continuous and discontinuous epitopes (overlayed) are shown in orange. For the L. *intermedia* PLD sequence, amino acid residues that are part of continuous epitopes are shown in green, while amino acid residues that are part of discontinuous epitopes are shown in blue. Amino acid residues that are part of both continuous and discontinuous epitopes (overlayed) are shown in purple. The order of antigenic epitopes is indicated with arrows and numbering. (For interpretation of the references to color in this figure legend, the reader is referred to the Web version of this article.)

Additionally, a comparison of the three-dimensional antigenic structure of the *L. intermedia* PLD consensus #1 sequence and two *L. laeta* PLD consensus sequences, belonging to *L. laeta* intra-specie clusters 1 and 2, revealed that the *L. laeta* PLD sequences (belonging to the *Loxosceles* inter-specie phylogenetic cluster 2) and the *L. intermedia* PLD sequence (belonging to the *Loxosceles* inter-specie phylogenetic cluster 1) differed in the location of the nine continuous epitopes that were found (Fig. 6). Also, although the PLD consensus 1 of the intra-species cluster 2 of *L. laeta* and the PLD consensus 1 of *L. intermedia* could be considered as class II PLDs, since both present two disulfide bridges, one in the catalytic loop and the other connecting the catalytic loop and the flexible loop, the antigenic structure differs slightly. Also, most of the predicted discontinuous epitopes were part of a previous predicted continuous epitope. However, one discontinuous epitope was identified in one of the active site-flanking loops (catalytic loop) of the protein, which is replicated in both *Loxosceles* species belonging to different inter-specie clusters, which this discontinuous epitope could be considered as a constant among the *Loxosceles* PLDs.

## 4 Discussion

The goal of the current study was to predict the antigenic structure and conservation of the phospholipase D toxins found in *Loxosceles* spider venom. To carry this out, an extensive search for PLD amino acid sequences of all the species of the genus *Loxosceles* had to be conducted. To ensure that the largest number of sequences available in the database were included in the analysis, four separate searches using the keywords "Phospholipase D," "Sphingomyelinase D," "Dermonecrotic toxin," and "LoxTox Protein" were performed on the database of the National Center for Biotechnology Information (NCBI). These four keywords were taken into consideration because of their former association with phospholipases D; as sphingomyelinase D was the first enzymatic classification of these toxins (Forrester et al., 1981; Kurpiewski et al., 1981), while phospholipase D is considered the actually accepted nomenclature to these enzymes (Chaim et al., 2011; Gremski et al., 2020; Lajoie et al., 2013; Lee and Lynch, 2005). Also, these toxins are extensively reported as their dermonecrotic capacity in the *in vivo* models skin, being designated as dermonecrotic toxins (Gremski et al., 2020). Additionally, other nomenclatures for these enzymes, such as Loxnecrogin (Machado et al., 2005), LoxTox toxins (Kalapothakis et al., 2007), or SicTox toxins (Binford et al., 2009), were considered in the search with the keyword LoxTox protein, which also included all the sequences displayed as the term SicTox toxins. Thus, the PLD sequences included in the study considered the six species of *Loxosceles* (*L. arizonica*, *L. gaucho*, *L. intermedia*, *L. laeta*, *L. reclusa*, and *L. rufescens*) which are frequently reported as having medical or clinical significance. However, the NCBI database reveals a considerably lower representation of PLD amino acid sequences for the less well-studied or newly discovered species of *Loxosceles* spiders, and also with relevant species such as *L. gaucho* and *L. reclusa*; therefore, future research must be considered to overcome this significant knowledge gap.

Some *Loxosceles* species, like *L. intermedia*, *L. spinulosa*, *L. laeta*, *L. boneti*, and *L. arizonica*, exhibited significant variation in their PLD amino acid sequences. In contrast, the remaining species showed less variation in the amino acid sequences for the PLDs of each species. Furthermore, *L. spinulosa* was the most divergent of all, with its own and possibly ancestral clade compared to the other *Loxosceles* species, as was shown in the phylogenetic tree constructed from the PLD consensus sequences for each species. Also, *L. laeta*, *L. spinulosa*, and *L. intermedia* showed the higher PLD sequence variability, so a unique phylogenetic clustering and consensus sequence was more challenging to establish than the other *Loxosceles* species with low variation in their PLD sequences. This divergence of the PLD from *L. laeta* was previously reported by Olvera et al. (2006), who showed that *L. laeta* belongs to a different phylogenetic cluster apart from the other species of *Loxosceles* from South and North America (Olvera et al., 2006), and was also previously observed at a lesser grade by us when the phylogenetic tree of the LlPLD1 and LlPLD2 sequences was analyzed (Catalan et al., 2011). Also, as was reported for *Loxosceles* phylogeny using 28S, COI and 16S genes (Binford et al., 2008), *L. spinulosa* showed the more divergent phylogeny distribution between *Loxosceles* species, and similarly, as was shown in the constructed PLD phylogeny tree, the species belonging to the *L*. *reclusa* group showed a close relationship, being clustered in the inter-species cluster 1. However, in contrast to the combined 28S/COI/16S tree where *L. laeta* was closely related to the clade of the *L*. *reclusa* group, in our tree it was possible to observe the diversity of the different *L. laeta* PLD consensus sequences. Thus, comparatively, the five consensus PLD sequences of *L. laeta* identified and used in the phylogenetic tree analysis for the PLDs of the genus *Loxosceles* appear in the least phylogenetic cluster composed of less related sequences, which is consistent with phylogenetic cluster IV of the alpha clade previously reported (Binford et al., 2009). Additionally, the distribution of the two sequences examined in the phylogenetic tree for *L. boneti* aligns with the distribution previously reported, with one sequence belonging to the α-clade and the other to the β-clade. Otherwise, the wide diversity of *L. intermedia* sequences that do not cluster together may point to the presence of paralogous rather than homologous genes. This particular distribution of *L. intermedia* PLD sequences has also been noted in the phylogeny of the Sicariidae family published previously (Binford et al., 2009).

About the antigenic epitopes, the linear epitope NLGANSIETDVSFDDNANPEYTYHGIP identified at the N-terminal region of the recombinant protein recLiD1 of *L. intermedia* could be regarded as a relevant epitope in *Loxosceles* PLD because it was able to produce an anti-recLiD1 serum with protective antibodies against lethal and dermonecrosis effects in mice treated with *L. intermedia* venom (Felicori et al., 2006). Also, serums produced against this peptide demonstrated cross-detection against the venoms of *L. gaucho*, *L. laeta* (Brazil), and *L. laeta* (Peru) (Dias-Lopes et al., 2010). Our analysis showed that this epitope is a common epitope region for the PLD sequences of *Loxosceles* but is shorter than the one that was previously reported, with only 19 of the 27 amino acid residues of the peptide. However, this predicted peptide was more prevalent in the PLDs from *Loxosceles* species that were included in the inter-specie phylogenetic cluster 1, consisting of closely related PLD sequences with high amino acid sequence identity where the PLD consensus 1 of the intra-specie cluster 1 of *L. intermedia* was present. Consequently, the conservation of this linear epitope dramatically decreases in the PLD sequences of *Loxosceles* belonging to the inter-specie phylogenetic cluster 2, where the PLD consensus 2 of the intra-specie cluster 2 of *L. intermedia* was present. Previously, the same epitope was identified at the 37–51 region of LiD1 sequence using distinct epitope prediction algorithms, including ElliPro, BepiPred, Kolaskar and Tongaonkar, and Emini (Buch et al., 2015). Nevertheless, sequence alignment analysis demonstrated that the epitope was not present in other *L. intermedia* isoforms, being absent from the LiRecDT3, LiRecDT5, and LiRecDT6 isoforms, but present in the LiD1, LiRecDT1, and LiRecDT2 isoforms (Buch et al., 2015). This finding corroborated the epitope's intra-specie variation of *L. intermedia* PLD isoforms. Moreover, in our study, the comparative identity percentage of the equivalent epitope #1-ANSIETDVSFDKQANPEYT with the sequences of PLD consensus 1 of *L. intermedia* (composed by LiD1, LiRecDT1, LiRecDT2, LiRecDT4, LoxTox i1, LoxTox i2, LoxTox i3, LoxTox i4, and LoxTox i5 sequences) showed 89.47 % of identity, and PLD consensus 2 of *L. intermedia* (composed by LiRecDT3, LiRecDT5, LiRecDT6, LiRecDT7, and LoxTox i6, LoxTox i7 sequences) showed only 31.58 % of identity, which was consistent with the findings reported by others. Furthermore, when epitope#1 was searched at consensus PLDs from *L. laeta*, the region was found between epitope regions 1 and 2 of the consensus sequences from *Loxosceles* phylogenetic inter-specie cluster 2, and the identity percentage was lower in the five consensus PLD sequences, ranging from 36.8 to 63 %. Therefore, even though it is a common epitope that can produce antibodies that can cross-detect with other *Loxosceles* venoms, it is more frequent in conserved PLD sequences of phylogenetically related *Loxosceles* species and varies significantly in sequences of PLD isoforms of relevant species like *L. intermedia* and *L. laeta*.

Subsequently, also in the LiD1 sequence, the antigenic epitope SKKYENFNDFLKGLR (residues 58–72) was identified using the strategy of identifying presumptive epitopes by detention with the polyclonal anti-recLiD1 serum (Felicori et al., 2009). About this epitope, part of this was also identified in our study, but it includes only the four amino acids (underlined) of the epitope#2 KGLRKATTPGDSKYHEKL identified from PLD sequences of the inter-specie phylogenetic cluster 1. In another study this peptide was only partially identified by two different predictors, Emini and Ellipro, but not by predictors Kolaskar and BepiPred (Buch et al., 2015). In our study, for the prediction of linear antigenic epitopes for B-cells, we used the BepiPred 2.0 server, which has the advantage over other predictors and the BepiPred 1.0 version, because it provides higher-quality and more accurate predictions for linear antigenic epitopes for B cells than other prediction tools, and the epitope data delivered are derived from crystallized protein structures (Jespersen et al., 2017). Subsequently, further evaluation of the 15-mer of LiD1 by mouse serum detected the peptide GLRSATTPGNSKYQEKLV as an epitope (Oliveira et al., 2016), which was concordant (underlined) with the epitope#2 KGLRKATTPGDSKYHEKL identified by us. Also, the authors reported the epitope AAYKKKFRVATYDDN from the C-terminal region of LiD1 (Oliveira et al., 2016). This antigenic region was homologous to the one identified as a common epitope#9 KFRIATYEDNPWET from *Loxosceles* inter-specie cluster 1 and epitope#9 FRLATYDDNPWEKF from *Loxosceles* inter-specie cluster 2 identified in our study, with light residue modification; however, and importantly, it corroborates that this c-terminal region is a relevant antigenic region in PLDs. Regarding the other sequences found by (Felicori et al., 2009), we only identified the epitopes ANSIETDVSFDKQANPEYT (previously reported peptide IETDVSFDDNANPEY) and MDKVGYDFSGNDDIGDV (previously reported peptide DKVGHDFSGNDDISD) as antigenic epitopes or as part of an epitope domain. Also, when the epitope SKKYENFNDFLKGLR was searched in the different consensus sequences of PLD for *L. laeta*, the identity percentage was low, from 38.9 % up to 61 %. Thus, this epitope domain was replaced in PLDs from *L. laeta* by a species-specific sequence, which indicates that the epitope sequence depends on the species and is not a common epitope for the genus *Loxosceles* in evolutionarily distant species.

Other antigenic epitopes have been reported from the sequence of SMase I from *L. laeta* where the peptides DNRRPIWNLAHMVNA (residues 2–16) and DFSGPYLPSLPTLDA (residues 164–178) were identified, and from A1H-LoxGa from *L. gaucho* the epitope EFVNLGANSIETDVS (residues 22–36) was identified (Ramada et al., 2013). From these, the epitope DNRRPIWNLAHMVNA was identified only partially (underlined) in PLD sequences of *L. laeta*, *L. intermedia* and *L. similis*, and could be considered a specific epitope for the three species. However, the epitope was absent in sequences belonging to the *Loxosceles* inter-specie phylogenetic cluster 1. About the peptide EFVNLGANSIETDVS, it was already identified as part of the epitope#1-ANSIETDVSFDKQANPEYT from the inter-specie phylogenetic cluster 1. Furthermore, it was found that the peptide DFSGPYLPSLPTLDA was not present at any of the *L. laeta* PLD consensus sequences but rather was partially identified as a component of epitope #6- MDKVGYDFSGNDDIGDV at sequences belonging to the *Loxosceles* inter-specie phylogenetic cluster 1. Additionally, it was partially present (underlined) at epitope #6-EKIGWDFSGNEDLGDI in sequences that belonged to inter-specie phylogenetic cluster 2 of *Loxosceles* sequences. Therefore, we investigated if this peptide was present in other PLDs from *L. laeta*, since the epitope DFSGPYLPSLPTLDA was previously identified at SMase I of *L. laeta* (Ramada et al., 2013), and it was a sequence included at the phylogenetic intra-specie cluster 1 from *L. laeta*. Thus, it was only present, including the residues PYLPSLPT, in the more conserved PLD sequences of *L. laeta*, but not in PLD sequences of *L. laeta* with greater diversity. Furthermore, other *Loxosceles* species do not contain the peptide PYLPSLPT. The latter supported the *Loxosceles* genus's significant diversity of antigenic epitopes at PLD sequences. About the discontinuous epitope prediction in PLD from *Loxosceles*, only was possible to evaluate the ones from species *L. laeta* and *L. intermedia*, because of the availability of three-dimensional structure, however, it was possible to compare the conformational antigenic structure of representatives sequences of phylogenetic inter-specie cluster 1 (*L. intermedia* PLD consensus 1) and cluster 2 (*L. laeta* PLD consensus 1 intra-specie cluster 1 and *L. laeta* PLD consensus 1 intra-specie cluster 2). The based tridimensional structure used as templates for modeling the PLD consensus sequences were the SMase I from *L. laeta* (Murakami et al., 2005), and Phospholipase D class II from *L. intermedia* (de Giuseppe et al., 2011). The first of them is representative of the class I of PLD, and the second represents a class II PLD, according to the structural classification proposed by Murakami et al., 2005), where the PLD from class I are characterized to possess a single disulphide bridge and an extended hydrophobic loop, while que PLD from class II are characterized to possess an additional intra-chain disulphide bridge linking the flexible loop and the catalytic loop (Murakami et al., 2005; de Giuseppe et al., 2011). Thus, a relevant discontinuous epitope was identified in amino acid residues from the catalytic loop independently of the class and phylogenetic cluster to which the PLD belongs.

Thus, the amino acid variation of sequences from the PLD protein family has implications not only for the documented differences at the level of phospholipase activity and variation in the lesion caused but also could significantly influence the antigenic structure considering our analysis and results. Also, considering that other authors have also identified previously possible sequences as epitopes but only a few of them showed immunogenic potential to produce and to be detected by immune serum (Felicori et al., 2009), it is necessary to evaluate the antibody production capacity of each of the nine predicted epitopes in order to experimentally determine their applicability for biotechnological purposes.

The significance of mapping the antigenic epitopes from PLDs of *Loxosceles* has allowed the use of these epitopes to produce neutralizing antibodies from single epitopes or as part of non-toxic chimeric proteins with multiple epitopes, such as the chimeric antigen formed by epitopes from LiD1 of *L. intermedia*, NLGANSIETDVSFDDNANPEYTYHGIP, SKKYENFNDFLKGLR, and NCNKNDHLFACW, and the epitopes DFSGPYLPSLPTLDA, and DLGANALEADVTFKGSVPTYTYHGTP from *L. laeta* SMase I (Ramada et al., 2013; Souza et al., 2018). Nonetheless, considering the broad diversity of amino acid sequences found in the family of phospholipase D toxins produced by *Loxosceles* spiders, as well as the significant intra-species variation found in the medically significant species *L. laeta* and *L. intermedia*, it is essential to consider the use of multi-epitope chimeric proteins rather than single antigenic epitopes or single recombinant proteins as immunogens for the production of antibodies with therapeutic potential. These could include highly immunogenic PLD epitopes from various *Loxosceles* species as well as the use of epitopes from other *Loxosceles* venom toxins, as reported by Lima et al. (2018), who created the recombinant multiepitopic protein derived from loxoscelic toxins (rMEPLox) by combining a linear epitope of sphingomyelinase D from SMase I from *L. laeta* and linear epitopes of hyaluronidase from *L. intermedia* with the formerly quimeric protein (rCpLi) (Mendes et al., 2013), which showed *in vivo* and *in vitro* neutralization of sphingomyelinase, hyaluronidase, and metalloproteinase activity of *L. intermedia* venom (Lima et al., 2018). In addition, this multiepitopic protein was tested to provide *in vivo* protection against *L. laeta* venom; however, this protection was only partial (Dantas et al., 2016), which suggests that these multi-epitope chimeric proteins still do not have an extensive enough antigenic spectrum to elicit an immune response similar to that of sera raised against the whole venom. Additionally, it could be explained since the *L. laeta* PLD epitope used was not common and not representative of the PLD diversity. Nevertheless, our findings indicated that PLDs from *L. laeta* exhibited the highest antigenic variability amongst *Loxosceles* species, suggesting that a strategy involving a combination of multiepitopic proteins formed by multiple conserved epitopes of PLD and other venom toxins specific to each of the most variable and medically relevant species (*L. laeta*, *L. intermedia*, and *L. gaucho*) must be considered. This would improve the protective effects and also have the potential to be a broad vaccine. Also, the use of recombinant mutated phospholipases D at essential amino acid residues involved in catalysis, binding to substrates, and magnesium-ion coordination could be considered as another possibility for a potential non-toxic vaccine, as was reported by Polli et al., 2021, who demonstrated that a combined vaccination protocol of mutated PLD from *L. intermedia*, *L. laeta*, and *L. gaucho* elicited a protective response to the venoms in rabbits (Polli et al., 2021).

Finally, the antigenic variations found in this antigenic epitope predictive analysis for the phospholipase D toxin family of the different species of *Loxosceles* spiders represent a biotechnological challenge when these toxins are potentially employed as antigens for monoclonal antibody production for diagnostic purposes. This could occur because a specific monoclonal antibody developed to detect an antigenic epitope of a PLD isoform from the venom of a particular *Loxosceles* spider species, such as *L. laeta*, may have low detection capacity for antigenic epitopes present in another PLD isoform from the same species but with high variability of amino acid sequences, as well as in *Loxosceles* species phylogenetically distantly related to *L. laeta*. This means that a diagnostic assay for loxoscelism detection, such as a sandwich ELISA or lateral flow immunochromatography assay (LFIA), must necessarily include multiple sets of antibodies in their conjugate and detection zones capable of detecting multiple PLD isoforms from the venom of different *Loxosceles* species. This would complicate the assay's setup due to the number of other inputs (monoclonal antibodies) required for its assembly, which would also raise the assay's production costs and the ultimate market price of the product. Therefore, it appears unlikely that a single or "universal" diagnostic test for determining the *Loxosceles* genus will exist and be applicable in multiple geographic regions. Instead, the best course of action appears to be the development of a diagnostic test that will be applicable in a region where cases of loxoscelism are primarily caused by a specific *Loxosceles* species.

## CRediT authorship contribution statement

**Alejandro Catalán:** Writing – review & editing, Writing – original draft, Supervision, Resources, Methodology, Investigation, Funding acquisition, Data curation, Conceptualization. **Carolina García:** Investigation, Formal analysis. **Valentina Sambra:** Investigation, Formal analysis. **Nicole Cadena:** Investigation, Formal analysis. **José Rojas:** Writing – original draft, Project administration. **Tomás Arán-Sekul:** Writing – original draft, Project administration. **Juan San Francisco:** Software, Resources, Data curation. **Valeria Vásquez-Saez:** Project administration. **Christian Muñoz:** Validation, Resources, Funding acquisition, Conceptualization. **Abel Vásquez:** Writing – review & editing, Validation, Resources. **Jorge E. Araya:** Resources, Project administration, Funding acquisition, Conceptualization.

## Ethical statement

This work does not involve any research on humans or animals and is therefore not conducted with any specific institutional ethical approval.

## Funding

This study was supported by 10.13039/501100002848CONICYT + 10.13039/501100008736FONDEF/CONCURSO IDeA I + D, 10.13039/501100008736FONDEF/10.13039/501100002848CONICYT 2020 N°ID20I10056 from Agencia Nacional de Investigación y Desarrollo (10.13039/501100020884ANID), Chile.

## Declaration of competing interest

The authors declare the following financial interests/personal relationships which may be considered as potential competing interests:Alejandro Catalan reports financial support was provided by Agencia Nacional de Investigacion y Desarrollo (10.13039/501100020884ANID). Alejandro Catalan has patent MÉTODO INMUNOCROMATOGRÁFICO RÁPIDO PARA LA DETECCIÓN TEMPRANA DEL LOXOSCELISMO pending to 202403632. If there are other authors, they declare that they have no known competing financial interests or personal relationships that could have appeared to influence the work reported in this paper.

## Data Availability

Data will be made available on request.
