# Supplementary material for: Predictive analysis of B-cell antigenic epitopes in phospholipase D toxins from Loxosceles spiders
Source: Toxicon X. 2025 Mar 26;26:100222. doi: 10.1016/j.toxcx.2025.100222 (PMC11994341; doi:10.1016/j.toxcx.2025.100222)
Supplement: Multimedia component 1 [file mmc1.docx]

**Appendix A. Supplementary figures**

**Manuscript**: Predictive analysis of B-cell antigenic epitopes in phospholipase D toxins from *Loxosceles* spiders


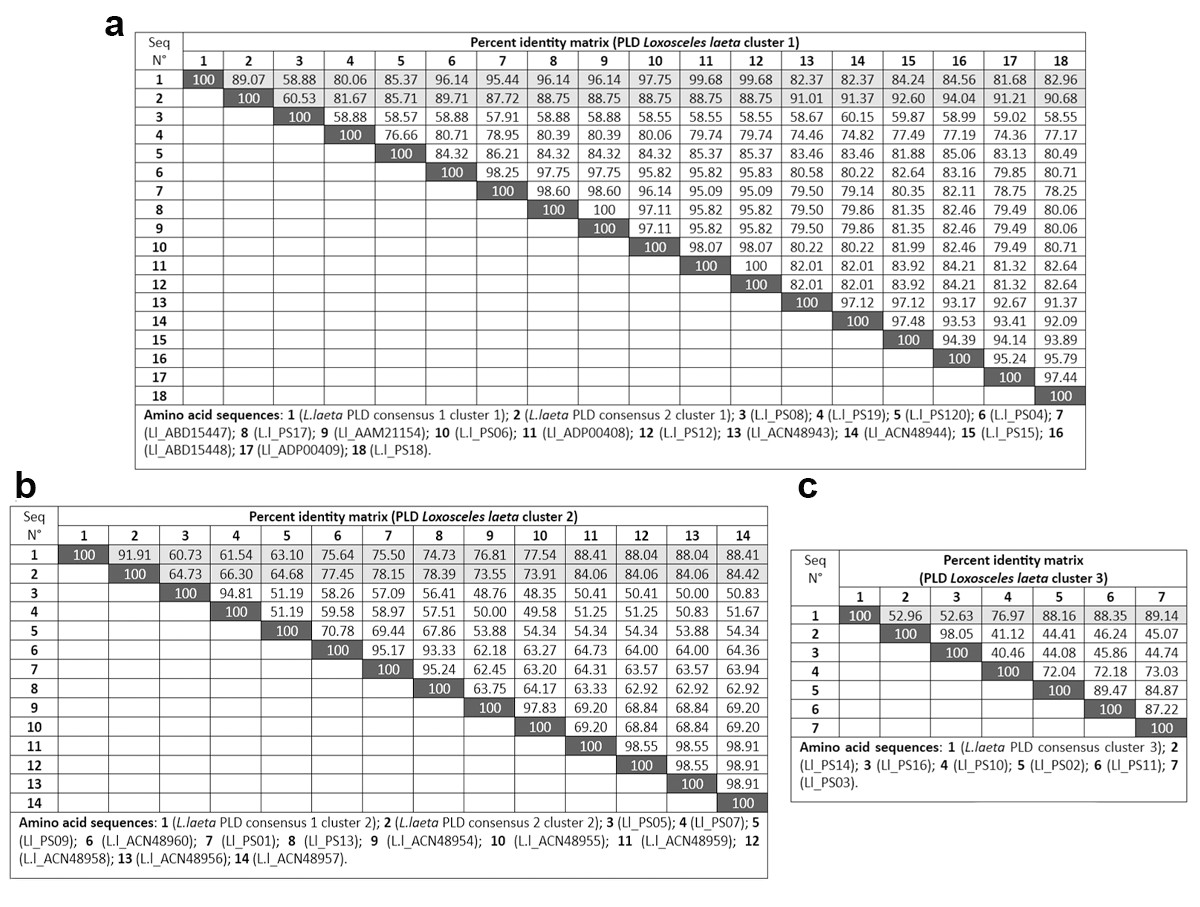


**Supplementary figure 1**. Identity matrix tables for phospholipases D from *Loxosceles laeta*. **a)** Percent identity matrix of aminoacidic sequences of phospholipases D from *L. laeta* belonging to the intra-specie cluster 1. Percent identities were obtained using the Clustal Omega multiple sequence alignment software v.1.2.4. for 16 seq with >70% identity. Access number for amino acid sequences are listed as table foot. Light gray: Percent identity of PLD consensus sequences vs single PLD sequences. **b)** Percent identity matrix of aminoacidic sequences of phospholipases D from *L. laeta* belonging to the intra-specie cluster 2. Percent identities were obtained using the Clustal Omega multiple sequence alignment software v.1.2.4. for 12 sequences with 48 up to 70% identity. Access number for amino acid sequences are listed as table foot. **c)** Percent identity matrix of aminoacidic sequences of phospholipases D from *L. laeta* belonging to the intra-specie cluster 3. Percent identities were obtained using the Clustal Omega multiple sequence alignment software v.1.2.4. for 6 sequences with <45% identity. Access number for amino acid sequences are listed as table foot.


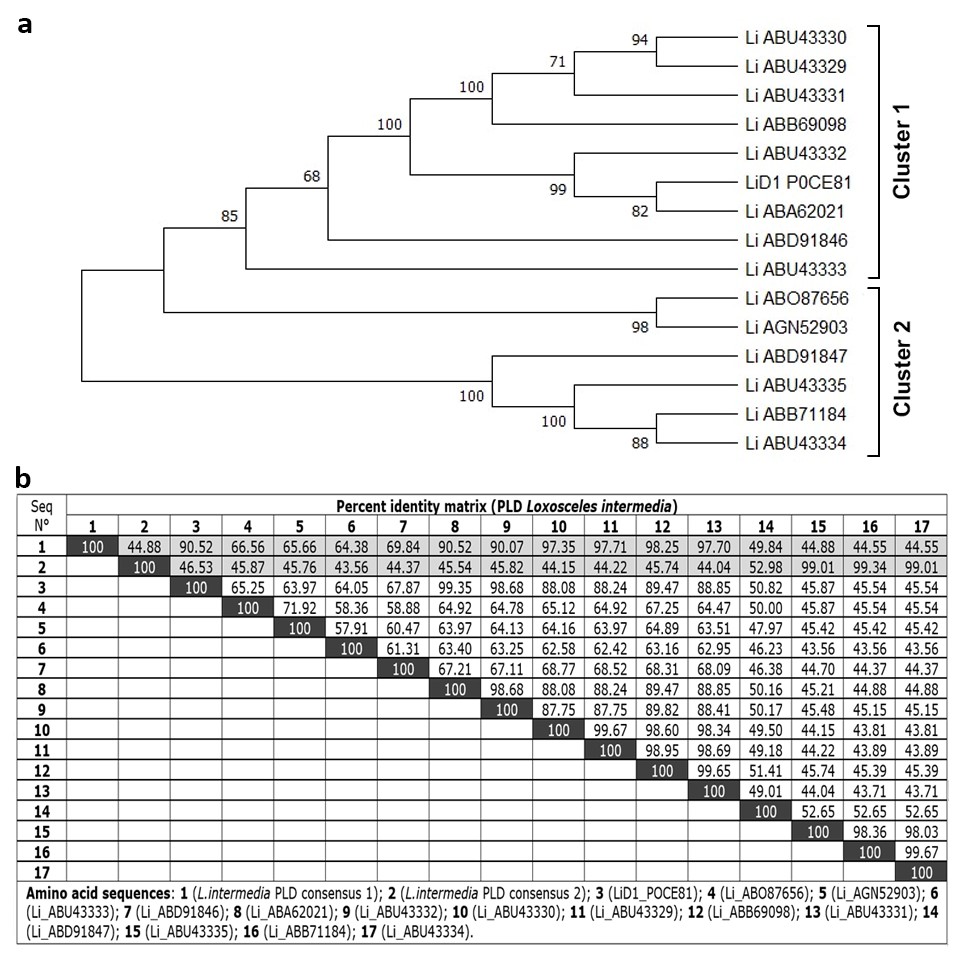


**Supplementary figure 2**. Phylogenetic analysis of phospholipases D from *Loxosceles intermedia*. **a)** Maximum likelihood phylogenetic tree of aminoacidic sequences of phospholipases D from *L. intermedia*. The tree was inferred by using the Maximum Likelihood method and Whelan and Goldman model, and the tree with the highest log likelihood (-3786.50) is shown. The percentage of trees in which the associated taxa are clustered together is displayed next to the branches. A discrete Gamma distribution was used to model evolutionary rate differences among sites (5 categories (+G, parameter = 2.0220)). This analysis involved 14 amino acid sequences. There were a total of 313 positions in the final dataset. Evolutionary analyses were conducted in MEGA X. **b)** Percent identity matrix of aminoacidic sequences of phospholipases D from *L. intermedia.* Light gray: Percent identity of PLD consensus sequences vs single PLD sequences. Percent identities were obtained using the Clustal Omega multiple sequence alignment software v.1.2.4. for 17 sequences. Access number for amino acid sequences are listed as table foot.


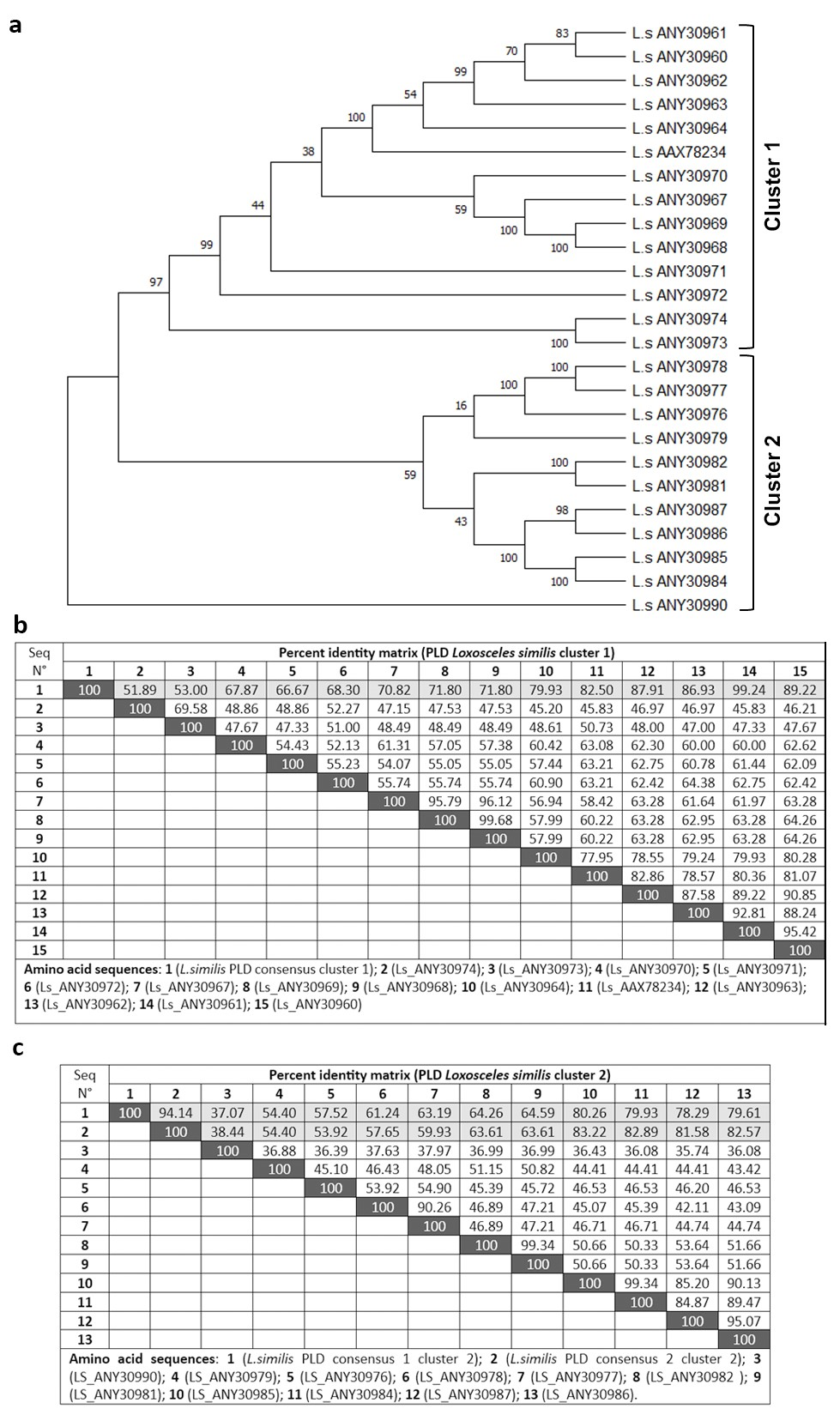


**Supplementary figure 3**. Phylogenetic analysis of phospholipases D from *Loxosceles similis*. **a)** Maximum likelihood phylogenetic tree of aminoacidic sequences of phospholipases D from *L. similis*. The tree was inferred by using the Maximum Likelihood method and Whelan and Goldman model, and the tree with the highest log likelihood (-8115.34) is shown. The percentage of trees in which the associated taxa clustered together is displayed next to the branches. A discrete Gamma distribution was used to model evolutionary rate differences among sites (5 categories (+G, parameter = 2.4640)). The rate variation model allowed for some sites to be evolutionarily invariable ([+*I*], 5.65% sites). This analysis involved 25 amino acid sequences. There were a total of 372 positions in the final dataset. Evolutionary analyses were conducted in MEGA X. **b)** Percent identity matrix of aminoacidic sequences of phospholipases D from *L. similis* belonging to the intra-specie cluster 1*.* Light gray: Percent identity of PLD consensus sequences vs single PLD sequences. Percent identity matrix of aminoacidic sequences of phospholipases D from *L. laeta*. Percent identities were obtained using the Clustal Omega multiple sequence alignment software v.1.2.4. for 14 sequences with >47% identity. Access number for amino acid sequences are listed as table foot. **c)** Percent identity matrix of aminoacidic sequences of phospholipases D from *L. similis* belonging to the intra-specie cluster 2*.* Light gray: Percent identity of PLD consensus sequences vs single PLD sequences. Percent identity matrix of aminoacidic sequences of phospholipases D from *L. laeta*. Percent identities were obtained using the Clustal Omega multiple sequence alignment software v.1.2.4. for 11 sequences with <47% identity. Access number for amino acid sequences are listed as table foot.
